# Assessing the Potential Interactions between Cellular miRNA and Arboviral Genomic RNA in the Yellow Fever Mosquito, *Aedes aegypti*

**DOI:** 10.3390/v11060540

**Published:** 2019-06-10

**Authors:** Pei-Shi Yen, Chun-Hong Chen, Vattipally Sreenu, Alain Kohl, Anna-Bella Failloux

**Affiliations:** 1 Unit of Arboviruses and Insect Vectors, Department of Virology, Institut Pasteur, 75015 Paris, France; 2 National Health Research Institutes, National Institute of Infectious Diseases and Vaccinology, Miaoli 35053, Taiwan; chunhong@nhri.org.tw; 3 National Mosquito-Borne Diseases Control Research Center, Miaoli 35053, Taiwan; 4 MRC-University of Glasgow Centre for Virus Research, Glasgow G61 1QH, Scotland, UK; Sreenu.Vattipally@glasgow.ac.uk (V.S.); Alain.Kohl@glasgow.ac.uk (A.K.)

**Keywords:** *Aedes aegypti*, arboviruses, chikungunya, dengue, Zika, miRNA

## Abstract

Although the role of exogenous small interfering RNA (siRNA) and P-element induced wimpy testis (PIWI)-interacting RNA (piRNA) pathways in mosquito antiviral immunity is increasingly better understood, there is still little knowledge regarding the role of mosquito cellular microRNA (miRNA). Identifying direct interactions between the mosquito miRNAs and the RNA genome of arboviruses and choosing the relevant miRNA candidates to explore resulting antiviral mechanisms are critical. Here, we carried out genomic analyses to identify *Aedes aegypti* miRNAs that potentially interact with various lineages and genotypes of chikungunya, dengue, and Zika viruses. By using prediction tools with distinct algorithms, several miRNA binding sites were commonly found within different genotypes/and or lineages of each arbovirus. We further analyzed those miRNAs that could target more than one arbovirus, required a low energy threshold to form miRNA-viralRNA (vRNA) complexes, and predicted potential RNA structures using RNAhybrid software. We predicted miRNA candidates that might participate in regulating arboviral replication in *Ae. aegypti*. Even without any experimental validation, which should be done as a next step, this study can shed further light on the role of miRNA in mosquito innate immunity and targets for future studies.

## 1. Importance

The role of the small interfering RNA (siRNA) and P-element induced wimpy testis (PIWI)-interacting RNA (piRNA) pathways in controlling virus infections in mosquitoes has been extensively studied and are considered to be a major part of the antiviral innate immune response. Although several studies have indicated that cellular microRNAs (miRNA) are involved in mosquito antiviral immunity, the miRNA-viralRNA (vRNA) direct interaction and its effect on virus replication in mosquitoes are still unclear. The cellular miRNAs of the mosquito *Aedes aegypti*, a vector for many arboviral diseases, may directly interact with three major arboviruses: chikungunya, dengue, and Zika viruses. By using the miRanda and TargetSpy tools (http://bioinfo5.ugr.es/srnatoolbox), several miRNAs were predicted to have potential binding sites that are common to multiple viral genotypes or lineages. Further analysis was carried out on miRNA-vRNA interactions that required a low energy threshold to form a complex. This study shows a broad picture of possible interactions between mosquito cellular miRNAs and the viral RNA of different genotypes/lineages of arboviruses, providing a list of mosquito cellular miRNAs candidates for experimental validations in future studies.

## 2. Introduction

Emerging and reemerging arthropod-borne viruses (arboviruses) are spreading globally in recent decades [1]. Arboviruses usually have RNA genomes, including positive-strand RNA alphaviruses (genus *Alphavirus*, family *Togaviridae*) and flaviviruses (genus *Flavivirus*, family *Flaviviridae*) [2]. The evolution of mosquito-borne RNA viruses and the complex interplay between the vector, the host, and the virus can shape arboviral emergence and re-emergence [3,4]. Infection of the arthropod midgut epithelial cells occurs following ingestion of a viremic blood meal; the ingested virus must disseminate through internal tissues and organs before reaching the salivary glands to be transmitted. Thus the virus has to overcome a series of tissue barriers before being secreted in mosquito saliva when it takes its next blood meal [5]. Each barrier has different tissue-specific immune properties, which, once triggered by viral infection, may affect the mosquito’s overall vector competence [6]. Immune responses to arboviruses involve different pathways, but key roles are played by small RNA/RNA interference (RNAi) pathways [7,8,9,10], which have been shown to be a major innate antiviral immune response in mosquitoes against arboviruses of all families [11,12]. 

Mosquitoes have three major types of small RNA pathways and associated molecules: the siRNA, piRNA, and miRNA pathways [9,11,12,13]. The siRNA antiviral immunity of mosquitoes was triggered by exogenous long double-stranded RNA (dsRNA) molecules, which are produced during viral replication. These dsRNAs are recognized by several siRNA biogenesis components and processed into predominantly 21 nucleotides (nt) mature viral siRNAs (vsiRNA). These vsiRNAs are loaded into the multi-protein RNA-Induced Silencing Complex (RISC), which contains the endonuclease Argonaute-2 (Ago-2). The viral RNA genome (or mRNA) containing complementary sequence could be targeted by Ago-2, trigger the cleavage of the target RNA and consequently, reduce the virus replication. These results have stressed the role of exogenous RNAi pathway in controlling viral replication [14,15,16,17,18].

Accumulating data suggest that the piRNA pathway is also involved in mosquito RNAi antiviral immunity [12,19,20,21,22,23], although the effector and effector mechanism are unclear. piRNAs are 24–30 nt in size and produced in a Dicer-independent manner. piRNA molecules interact with Argonaute-3 (Ago-3) and Piwi proteins in a so-called “ping-pong” mechanism which amplifies these small RNAs [24]. Several studies have identified and characterized viral piRNA (vpiRNA) or piRNA-like small RNAs in mosquitoes or mosquito cells [12,20,21,22,23]. Nonetheless, it remains unclear if vpiRNAs exert an antiviral activity.

Both siRNA and piRNA antiviral immunity could be triggered by the direct binding of small RNA on the viral RNA genome. Some studies indicated that the miRNA pathway could presumably participate in antiviral responses [25,26,27,28,29,30,31]. The miRNA pathway shares some similarities with the siRNA pathway, but their differences are more significant [9,11,32]; both have nuclear and cytoplasmic phases as the precursor RNAs are produced in the nucleus. The miRNA precursors (pri-miRNAs) originate from independent miRNA genes or mirtrons which are encoded as introns within RNA transcripts. The hairpin-structured pri-miRNAs are processed by the RNase III-type endonuclease Drosha into ~70 bp hairpins, which are then cleaved into ~20 bp miRNA duplexes by Dicer-1 after being exported from the nucleus into the cytoplasm by Exportin5 [33,34,35]. The miRNA duplexes in the cytoplasm are loaded into Ago-1 or Ago-2 proteins in miRNA-induced silencing complexes (miRISCs) according to their different structures [36,37,38]. Mainly using residues 2–8 at the 5′ end of mature miRNA (seed region), the miRISC uses the guide strand to find complementary RNA sequences, which leads to RNA degradation (carried out by Ago-2), translational inhibition, or both (mediated by other Ago proteins other than Ago-2) [39,40]. Commonly, animal miRNA binding sites are mainly in the 3′UTR [41], however miRNA binding sites in the 5′UTR or coding regions were also reported and are involved in post-transcriptional gene regulation [42,43,44]. The miRNA antiviral immunity in mosquitoes was shown to facilitate the replication of dengue virus (DENV) or West Nile virus (WNV) in mosquitoes or *Drosophila* cell lines that are deficient in the biogenesis of some miRNA components [45,46]. In addition, the miRNA transcriptomes were also changed in response to chikungunya virus (CHIKV), DENV, and Zika virus (ZIKV) infection in *Aedes spp* [29,31,47,48,49,50].

Mosquito miRNAs are involved in viral replication by regulating host factors or direct miRNA-vRNA interaction. Some miRNAs reported in *Aedes spp.* mosquitoes can limit viral replication as a consequence of the down-regulation of mosquito genes. A miRNA, miR-2940 in *Ae. aegypti* and *Aedes albopictus* could be triggered by virus infection leading to a down-regulation of metalloprotease, which is essential for virus replication. This antiviral miRNA inhibits the replication of CHIKV, DENV, WNV, and even Palm Creek virus (PCV, an insect-specific flavivirus) in *Ae. aegypti* or *Ae. Albopictus* [30,48,51,52]. Notably, even though bioinformatics approaches suggested a target site of miR-2940 in the 3‘UTR of WNV, it has been experimentally demonstrated that this miRNA has no significant effect on viral replication [52]. Another example is aae-miR-2b-3p in *Ae. aegypti*; aae-miR-2b-3p is up-regulated in response to CHIKV infection. Knocking down an ubiquitin-related modifier (URM) [53] impedes the thiolation of tRNA and consequently, limits CHIKV infection [54].

Surprisingly, only a few studies have shown that mosquito miRNAs interfere with virus replication via complementarity with viral RNA genome in mosquitoes. Two binding sites of aal-miR-252 and aal-miR-281 (from *Ae. albopictus*) were found in the genomic RNA of DENV at the envelope protein and 5′UTR regions, respectively [55,56]. However, aal-miR-252 can limit the viral replication, whereas aal-miR-281 facilitates DENV replication via a direct interaction at the 5′UTR. Besides, expressing synthetic miRNAs complementary to arboviral RNA genome was shown to reduce the transmission of CHIKV, DENV, and ZIKV [57,58], thus indirectly supporting the hypothesis that cellular miRNA can target viral nucleic acids and replication.

There is still a remarkable lack of information about the direct miRNA-vRNA interaction in mosquitoes. However, miRNAs are more easily identified by next-generation sequencing (NGS) techniques and are now available in databases while improved bioinformatics tools have been developed. To predict potential mosquito miRNA binding sites in the viral RNA, we compared published small RNA sequencing data from *Ae. aegypti* to virus sequence data of three major arboviruses, e.g., CHIKV, DENV (serotypes 1–4), and ZIKV. We predicted and analyzed the potential target sites on each virus genome to reveal practicable miRNA-vRNA interactions by combining thermodynamics and miRNA expression profiles. This approach can underpin future studies on the role of miRNAs in regulating arbovirus replication in mosquito cells. Notably, the biological meaning of the prediction results was only important after experimental validations.

## 3. Methods

### 3.1. Identification Strategies for miRNA and vRNA Interactions

Key human pathogenic arboviruses (flaviviruses, DENV1–4, ZIKV, and the alphavirus, CHIKV) were chosen for analyzing the relationship between miRNAs and viral genomes (vRNA). The genome sequences for each virus were collected from the virus database Virus Pathogen Resource (ViPR, www.ViPRbrc.org) [59,60], while the miRNA sequences of *Ae. aegypti* were retrieved from the miRNA database, miRBase, and published results of small RNA sequencing [61]. Predictions of miRNA-vRNA interactions were carried out mainly using miRanda software [62] and in coordination with TargetSpy [63] via the online tool sRNAtoolbox [64] with default settings (http://bioinfo5.ugr.es/srnatoolbox). The consensus binding sites predicted by both software were extracted by BEDtools (version 2.25.0) [65]. Only the prediction sites shared by the two prediction algorithms were chosen for a further case study and evaluation of the affinity of each miRNA-vRNA complex. In addition, the structures of these complexes were predicted using the tool RNAhybrid [66] via BiBiServ2 (https://bibiserv2.cebitec.uni-bielefeld.de). Total of 261, 1671, 1244, 884, 164, and 157 complete viral genomes of CHIKV, DENV1–4, and ZIKV, respectively, were retrieved from ViPR as data input for miRNA-vRNA prediction; the viruses from each genotype were chosen for further analysis, as they showed the highest number of potential miRNA binding sites.

### 3.2. Flowchart Validation

Experiment data of a luciferase reporter of *Ae. aegypti* miRNA-mRNA interactions published by Zhang, et al. [67] were applied to validate the workflow for predicting miRNA binding sites we have adopted in this study. AAEL013070, AAEL006834, AAEL000577, and AAEL010015 of *Ae. aegypti* were shown to be regulated by aae-miR-11-3p (AAEL013070), aae-miR-275-3p (AAEL006834 and AAEL000577), and aae-miR-286b-3p (AAEL010015) in the 3′UTR [67]. Among the four most significant miRNA-mediated reductions validated by Zhang, Aksoy, Girke, Raikhel and Karginov [67], three of them could be correctly identified with our workflow. These transcripts and the miRNA database of *Ae. aegypti* were used as input data. Using the default setting, the 3′UTR binding sites for aae-miR-11-3p in AAEL013070 (−13.04 kcal/mol) and aae-miR-275-3p for AAEL006834 (−18.7 kcal/mol) and AAEL000577 (−15.89 kcal/mol), were commonly predicted by both miRanda and TargetSpy. Even though no consensus binding site at the 3′UTR for aae-miR-286b-3p could be found in AAEL000577 with both algorithms, a potential binding site for aae-miR-286b-3p at the 3′UTR could be detected at two distinct positions by miRanda and TargetSpy (http://bioinfo5.ugr.es/srnatoolbox).

## 4. Results

### 4.1. Several Potential miRNA Binding Sites Were Predicted in Viral RNA Genomes

**Figure 1:**
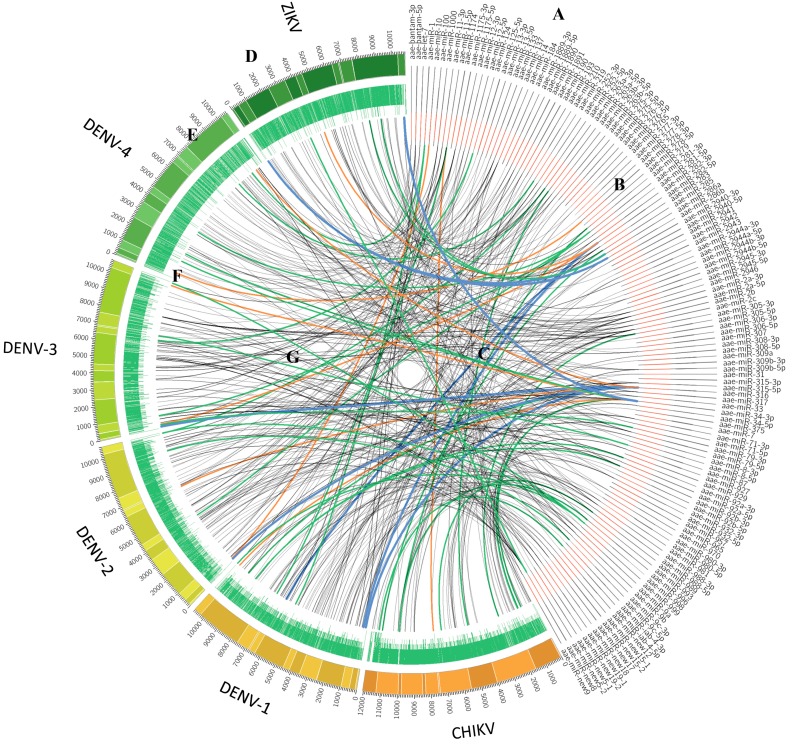
The interactome of *Ae. aegypti* micro RNAs (miRNAs) with chikungunya virus (CHIKV), Dengue virus (DENV), and Zika virus (ZIKV) genomes as predicted by miRanda and TargetSpy. (A) miRNAs of *Ae. aegypti*; (B) miRNA and its seed regions (seed regions in red); (C), links between miRNAs and potential binding regions on the viruses; (D) genomes of arboviruses; (E) dark and light areas represent the protein coding regions in each virus; (F) conservation scores of virus among all the genotypes; green links, the miRNA binding site shared among most of the genotypes discussed in this study; orange links, the miRNA-viralRNA (vRNA) with low energy; blue links, the miRNA-vRNA with low energy shared among most arboviruses; black links, remaining miRNA-vRNA interactions.

With our prediction flowchart, a total of 674 miRNA-vRNA interactions were predicted consensually by both algorithms for each virus (Figure 1). Among them, 93 potential binding sites could be found in CHIKV genome, 151, 130, 123, and 98 potential binding sites found in the genomes of DENV1–4, respectively, and 79 potential binding sites in the ZIKV genome.

### 4.2. miRNA Binding Sites in the CHIKV Genome

CHIKV belongs to the family of *Togaviridae* and the genus *Alphavirus*, with three genotypes circulating worldwide: East/Central/South African (ECSA), West African (WA), and Asian. One additional CHIKV lineage, Indian Ocean lineage (IOL), emerged in 2004–5 from the ECSA phylogroup and has spread throughout many tropical regions [68]. The IOL lineage predominates in regions where the vector *Ae. albopictus* is present/dominant, in part due to the selection of an *Ae. albopictus*-adaptive substitution in the CHIKV E1 envelope glycoprotein (E1-A226V). This substitution confers efficient infection and dissemination in *Ae. albopictus* for IOL of CHIKV [69,70]. The Asian and IOL/ECSA genotypes were responsible for the most recent outbreaks [71].

**Figure 2:**
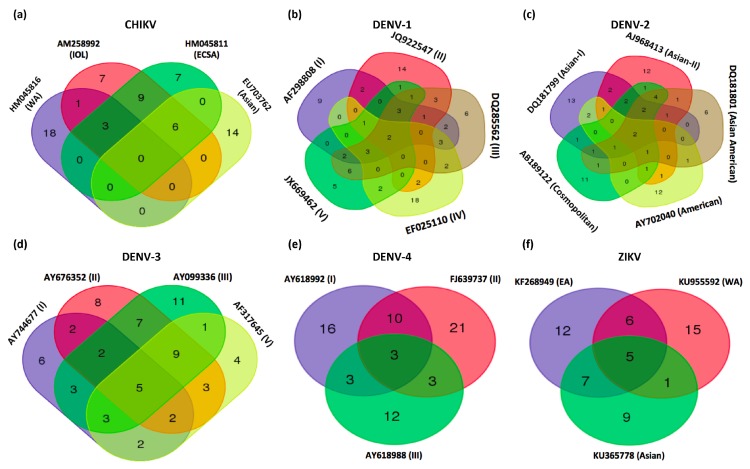
Venn-diagram presentation of common prediction binding sites on each genotype/lineage of CHIKV, DENV1–4, and ZIKV for *Ae. aegypti* miRNAs (**a**–**f**). miRNAs that were sorted by the conservation of target sites that might show a more general miRNA-vRNA interaction. (**a**) AM258992, Indian Ocean Lineage, IOL; HM045816, West African, WA; EU703762, Asian; HM045811, East/Central/South African, ECSA; (**b**) AF298808, genotype I; JQ922547, genotype II; DQ285562, genotype III; EF025110, genotype IV; JX669462, genotype V; (**c**) DQ181799, Asian-I; AJ968413, Asian-II; DQ181801, Asian American; AY702040, American; AB189122, Cosmopolitan; (**d**) AY744677, genotype I; AY676352, genotype II; AY099336, genotype III; AF317645, genotype V; (**e**) AY618992, genotype I; FJ639737, genotype II; AY618988, genotype III; (**f**) KF268949, East African, EA; KU955592, West African, WA; KU365778, Asian. The numbers with an asterisk represent the miRNA binding sites; the most common in each virus group were listed in Supplementary Table S1.

Four CHIKV genotypes were selected for this study: Asian (EU703762), ECSA (HM045811), IOL (AM258992), and WA (HM045816). Two analytical tools, miRanda and TargetSpy, were used to identify a total of 20, 25, 26, and 22 miRNA binding sites that were commonly found for CHIKV of different genotypes and lineages (Asian, ECSA, IOL, and WA) (Figure 2a).

When comparing the binding sites previously identified among all four genotypes, the highest number of shared binding sites was between ECSA and IOL genotypes (*n* = 18), whereas WA and Asian genotypes were relatively independent to each other with no common binding sites (Figure 2a). Among them, six miRNA binding sites were common among Asian, ECSA, and IOL genotypes, which could be targeted by aae-miR-263a-5p, aae-miR-279-3p, aae-miR-305-5p, aae-miR-34-3p, and aae-miR-996-3p (Supplementary Table S1). And among three potential miRNA binding sites that are commonly found among WA, ECSA, and IOL genotypes, two might potentially be targeted by aae-miR-285-5p and aae-miR-989-3p in the E1 and Capsid coding regions, respectively. The other miRNA binding site shared among WA, ECSA, and IOL genotypes may potentially be targeted by aae-miR-iab-4-5p in the 3′UTR. Notably, this miRNA might have an additional binding site on the 5′UTR of ECSA and IOL genotypes. Any activity of this miRNA might be increased by multiple binding sites on the 5′- and 3′ UTR of CHIKV involved in initiating viral RNA replication [72]. In addition, as the synthesis of subgenomic RNA from minus strand of RNA genome is critical for CHIKV replication, the minus strand genomic RNA of CHIKV was also analyzed via the flowchart. The result showed only one potential binding site that could be consensually found in the minus strand of CHIKV RNA genome; for aae-miR-282-5p (Supplementary Figure), the predicted binding sites were shown in Supplementary Table S2.

### 4.3. miRNA Binding Sites in DENV Genomes

DENV has evolved independently into four serotypes from distinct sylvatic progenitors and then into several genotypes [73]. They only share 60–75% sequence similarity and need to be analyzed separately to find potential miRNA-vRNA interactions [74]. Several conserved miRNA binding sites could be found in each serotype which might participate in viral regulation, as outlined below.

### 4.4. DENV-1

Five genotypes of DENV-1 were selected for analysis: genotype I (AF298808), genotype II (JQ922547), genotype III (DQ285562), genotype IV (EF025110), and genotype V (JX669462). A total of 28, 30, 34, 33, and 26 potential binding sites were found in genotypes I, II, III, IV, and V, respectively (Figure 2b). Among them, only two potential miRNA binding sites were common to all five genotypes of DENV-1, which could be targeted respectively by aae-miR-1-3p and aae-miR-282-5p on the capsid and NS3 protein coding regions (Supplementary Table S1). Furthermore, six potential miRNA binding sites were commonly found in four of the five genotypes (Supplementary Table S1). The NS5 region of genotypes I, II, III, and V could potentially be targeted by aae-miR-316-5p, aae-miR-92a-3p, and aae-miR-92b-3p. In addition, the miRNA aae-miR-316-5p might have a further potential binding site within the capsid region (genotypes I, III, IV, and V), which may increase the probability of miRNA-vRNA interactions. The only miRNA that was found with a common binding site in genotypes I, II, IV, and V within NS4B is aae-miR-11-5p (Supplementary Table S1). Two potential miRNA binding sites for aae-miR-263a-3p and aae-miR-998-3p were found conserved on the 3′UTR of genotypes I, III, and V (Supplementary Table S1).

### 4.5. DENV-2

Five genotypes of DENV-2 were selected for our analysis: Asian I (DQ181799), Asian II (AJ968413), Asian American (DQ181801), American (AY702040), and Cosmopolitan (AB189122). We found a total of 28, 31, 21, 22, and 28 potential binding sites on each genotype respectively (Figure 2c). Among them, binding sites for aae-miR-316-5p, and aae-miR-9c-5p were common to all five genotypes (Supplementary Table S1).

Three other miRNA binding sites were shared between at least four genotypes. Two binding sites were common between Asian I, Asian II, Asian American, and Cosmopolitan genotypes: aae-miR-281-3p within NS2B, and aae-miR-998-3p within the 3′UTR region of DENV-2. Another miRNA, aae-miR-315-5p, was also predicted to target the NS5 region of all DENV-2 genotypes except the Asian I genotype. The only miRNA predicted to target Asian I, Asian American, American, and Cosmopolitan genotypes of DENV-2 is aae-miR-263a-3p, which has a potential binding site on the 3′UTR. In addition, the same miRNA binding site within the 3′UTR of DENV-2 was also found within the same region of DENV-1.

### 4.6. DENV-3

Four genotypes of DENV-3 were selected for our analysis: genotype I (AY744677), genotype II (AY676352), genotype III (AY099336), and genotype V (AF317645). Again, 25, 38, 41, and 29 potential binding sites were identified in genotypes I, II, III, and V, respectively (Figure 2d). Five miRNA binding sites were predicted to be conserved in all four genotypes. Among these, aae-miR-124-3p, aae-miR-281-3p, and aae-miR-998-3p were predicted to target the NS4A, prM, and 3′UTR regions, respectively. Also, the capsid region was predicted to have two target sites for aae-miR-316-5p (except genotype I) and one target site for aae-miR-79-3p.

### 4.7. DENV-4

Three genotypes of DENV-4 were selected for our analysis: genotype I (AY618992), genotype II (FJ639737), and genotype III (AY618988). We identified 32, 37, and 21 potential miRNA binding sites for genotypes I, II, and III of DENV-4, respectively (Figure 2e). Among them, three potential miRNA binding sites were common to all three genotypes and targeted by aae-miR-1-3p, aae-miR-219-5p, and aae-miR-281-3p in NS5 and NS2A regions (Supplementary Table S1).

### 4.8. miRNA Targeting the ZIKV Genome

ZIKV was first isolated from *Aedes africanus* mosquitoes in 1948 [75] although serological evidence has shown a broader geographic distribution of human infections including North/East Africa and South/Southeast Asia [76,77,78,79,80,81,82,83]. Since the first human case reported in Nigeria in 1952 [81], only 13 cases of mild, febrile illness were reported until the outbreak in the State of Yap (Federated States of Micronesia) in 2007 [84,85,86,87], where more than 70% of the population were infected. [88] Later, cases of ZIKV related Guillain–Barré syndrome were notified during the outbreak in French Polynesia in 2013–2014. [89,90] The first ZIKV case reported in America was in 2015 in Bahia (Brazil) [91,92]. ZIKV caused a total of 51,473 suspected cases and more than 4300 cases of microcephaly in Brazil by March 2016 [93,94], and the virus spread to at least 33 countries or areas in the Americas [93,95]. Related to DENV, ZIKV belongs to the *Flaviviridae* family and the genus *Flavivirus*. The three distinct genotypes East Africa (EA), West Africa (WA), and Asian were likely to be originated in East Africa [96,97]. ZIKV has a conserved genome with less than 12% divergence at a nucleotide level among all virus strains, and with 99% nucleotide similarity for strains from the Americas [98]. Thus, the interactions between mosquito miRNA and viral RNA could be more relevant than between highly divergent viruses and might provide a new insight for evaluating the antiviral immunity of mosquitoes against newly emergent viruses. Three genotypes of ZIKV were selected for analysis: EA (KF268949), WA (JU955592), and Asian (KU365778). According to our results, 30, 22, and 27 potential binding sites were found in EA, WA, and Asian genotypes respectively (Figure 2f).

Five potential miRNA binding sites within the 5′UTR, NS2A, NS5, and 3′UTR were common among all three genotypes (Supplementary Table S1): aae-miR-286a-3p, aae-miR-286b-3p, aae-miR-34-3p, and aae-miR-new8. Among them, aae-miR-286a-3p, aae-miR-286b-3p, and aae-miR-34-3p were predicted to target the 5′ or 3′UTR region of ZIKV. Moreover, aae-miR-286a-3p has an additional potential binding site on the NS5 region.

### 4.9. Selection of miRNAs and Thermodynamic Analysis

**Figure 3:**
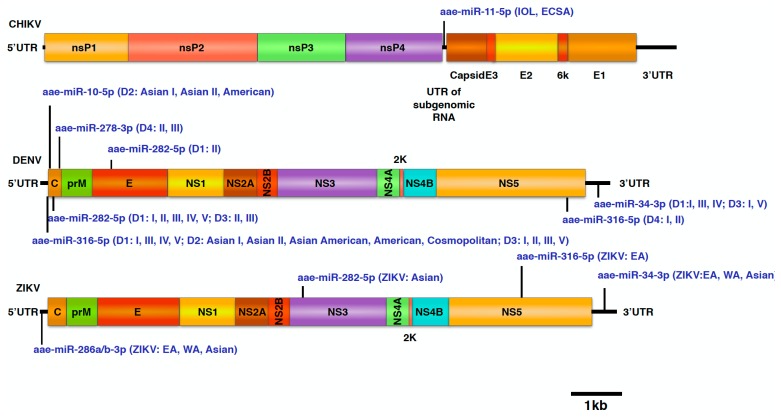
Predicted miRNA-vRNA interactions sorted with minimum free energy (MFE) value and compromised sequence conservation in each genotype/lineage in this study. The scheme shows the most likely miRNA-vRNA interactions predicted in this study. Each miRNA could target multiple viruses, has a relatively low MFE (around –20 kcal/mol), and is predicted to target more than one genotype of each virus.

**Figure 4:**
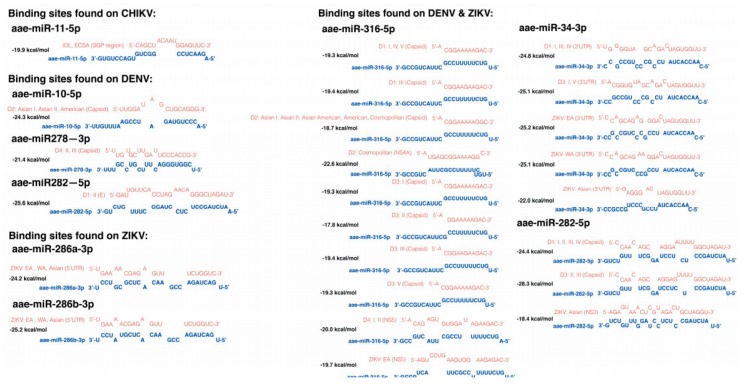
Possible structures of each low MFE miRNA-vRNA interaction predicted by RNAhybrid. The structures of seven low MFE miRNAs of *Ae. aegypti* potentially interact with CHIKV, DENV, and ZIKV. Among them, the possible binding sites for aae-miR-316-5p and aae-34-3p, and aae-miR-282-5p could be commonly found on both DENV and ZIKV. Sequence in red: the potential targets in RNA genome of each virus; sequence in blue: cellular miRNAs of *Ae. aegypti*.

Sequence complementary is an important but not the only factor that modulates the miRNA-vRNA interactions. The thermodynamic between miRNA and vRNA is another crucial factor that has an effect on the formation of miRNA-vRNA complex. Several miRNAs were predicted to have a low minimum free energy (MFE), indicating a relatively high affinity to form miRNA-vRNA complexes. To assess the potential interaction between miRNAs and the binding sites that are not highly conserved among genotypes but have relatively high potential to form miRNA-vRNA complexes in *Ae. aegypti*, we set up an MFE cut-off with –20 kcal/mol for evaluating miRNA-vRNA affinity [49,99]. According to this prediction data, we identified eight miRNAs—namely aae-miR-10-5p, aae-miR-11-5p, aar-miR-278-3p, aae-miR-282-5p, aae-miR-286a-3p, aae-miR-286b-3p, aae-miR-316-5p, and aae-miR-34-3p. These miRNAs could potentially target more than one genotype/lineage of each virus species with an MFE below –20 kcal/mol for each miRNA-vRNA complex (Figure 3). The possible structures formed by miRNA-vRNA interactions were predicted using RNAhybrid (Figure 4).

## 5. Discussion

As poikilotherms, the body temperature of mosquitoes is influenced by ambient temperature, resulting in a different miRNA thermodynamic stability profile in insects than vertebrates [100]. Using adequate algorithms that have high prediction accuracy in insects is then important for predicting the potential miRNA-vRNA interactions. Here, two algorithms, miRanda and TargetSpy, were chosen for analyzing the mosquito potential miRNA-vRNA interactions. Several potential mosquito miRNA-vRNA interactions could be consensually predicted by both algorithms. miRanda and TargetSpy are two powerful algorithms for predicting the miRNA potential binding sites on target sequences in insects. miRanda is one of the most extensively used miRNA target prediction tools and was applied originally for identifying miRNA binding sites in *Drosophila* and mosquitoes. The miRanda algorithm works in three phases. Firstly, the complementarity matches between the input miRNAs and RNA sequences were identified based on dynamic programming algorithm alignment. Secondly, a thermodynamic calculation was made to rule out the matches with an MFE value above the threshold. Finally, the remaining results were filtered by checking the sequence conservation with *Drosophila melanogaster*, *Drosophila pseudoobscura*, and *Anopheles gambiae*. In addition, miRanda can also weigh the matches between the 2^nd^ to the 8^th^ nucleotide from the 5′ arm of miRNA (seed-region), to evaluate the potential for that miRNA-vRNA complex to form [62]. For a more stringent selection, the same database was filtered with the other algorithm, TargetSpy. Unlike miRanda, TargetSpy is an algorithm based on machine learning and automatic feature selection with a broad spectrum of compositional, structural, and base pairing of each miRNA to the targeted sequence [63]. TargetSpy is able to predict species–specific targets, as the miRNA-vRNA interactions are not extensively studied. Besides, even though trained on mouse data, TargetSpy is an algorithm which shows the highest accuracy among several other algorithms on experimentally proven datasets (including insects) [63]. Using a combination of miRanda and TargetSpy, we obtained consensus results which should be more robust for detecting miRNAs that are interacting with viral genomes. Moreover, a more reliable miRNA-vRNA interaction was examined by comparing these results with the structure information predicted by RNAhybrid.

Despite the amount of false positive miRNA-target interactions predicted by several algorithms due to the nature of miRNA target recognition [101], other factors like MFE that affect miRNA-target interaction were applied for results sorting. The lower the MFE, the higher the potential to form the miRNA-target complex [102,103]; in order to narrow down the possible miRNA candidates, a relatively stringent cut-off, –20 kcal/mol, was applied in this study [62]. Even though MFE is an important factor affecting miRNA-vRNA complex formation, it does not certify that the interaction will lead to functional changes.

In this study, we found eight miRNA binding sites that both have low MFE and are also relatively conserved in each genotype/lineage. In theory, these miRNAs have a higher chance to form a miRNA-vRNA complex with more than one genotype/lineage of virus, and thus might subsequently be involved in virus replication. Moreover, according to the predicted binding sites, depending on the binding regions, some of them might have an even higher chance to participate in virus replication; the predicted binding sites for aae-mir-11-5p, aae-miR-286a/b-3p, aae-miR-316-5p, and aae-miR-34-3p are located in important viral regulatory regions such as the 3′UTR, 5′UTR, or the subgenomic RNA untranslated region of the three tested viruses. Aae-mir-286a/b-3p was predicted to target all genotypes of ZIKV at the 5′UTR; although not highly expressed, this miRNA is an ovary specific miRNA and could be deposited into embryos [104]. It might be worth exploring the potential relationship between this miRNA and the vertical transmission of ZIKV. Except for DENV-4, most DENV genotypes were predicted to have a binding site for aae-miR-316-5p at the 5′UTR region; aae-miR-316-5p being a miRNA that could be detectable in the fat body and slightly up-regulated in midguts 24 h after receiving a blood meal [67,105]. However, the effectiveness of aae-miR-316-5p might be reduced due to the relatively low expression level [105]. Conserved aae-miR-34-3p binding sites could be found in the 3′UTR of ZIKV, DENV-1, and DENV-3; this miRNA was reported to be up-regulated in response to CHIKV and *Wolbachia* infection [53,106]. Finally, the only miRNA that has low MFE and is relatively conserved among lineages of CHIKV is aee-miR-11-5p, which was predicted to target the end of the subgenomic untranslated RNA region, which also covers the start codon of the viral structural polyprotein of CHIKV. Therefore, the expression of CHIKV structural proteins might be influenced by the formation of a miRNA-vRNA complex at the end of subgenomic RNA untranslated regions, which might antagonize the binding region with host translational factors. In addition, aae-miR-11-5p is also one of the most abundantly expressed miRNAs in *Ae. Aegypti* [104], which could increase the chance of complex formation. Notably, the replication of the three arboviruses relies on the duplication of viral polyproteins. Thus, a single effective miRNA-vRNA interaction in the viral genome is sufficient to regulate viral replication. In mammals, more evidence is available on the role of virus replication regulated by direct miRNA-vRNA interactions [107,108,109,110,111,112,113,114,115], whether facilitation or reduction.

The critical roles of cellular miRNAs in host viral immunity have received more attention elsewhere with mammalian host miRNA-vRNA interactions [107,108]. In humans, the interaction between the liver specific miR-122 and the 5‘UTR of Hepatitis C virus (HCV) stabilizes the viral RNA facilitating viral replication [116,117]. On the contrary, the replication of the arbovirus Eastern equine encephalitis virus (EEEV) could be reduced by the human hematopoietic cell specific miR-142-3p that has four binding sites in the 3‘UTR [109]. In contrast, there is still a lack of information on the miRNA targetome for *Ae. aegypti.* However, few studies have demonstrated that the replication of some genotypes of DENV-2 could be affected by direct miRNA-vRNA interaction in *Ae. Albopictus* [55,56], and CHIKV, DENV-3, and ZIKV transmission could be reduced in synthetic antiviral miRNA expressing genetically modified *Ae. Aegypti* [57,58], suggesting the cellular miRNA can target viral RNA, and such interactions could potentially affect viral RNA replication.

In this study, we used the consensus results from two adequate algorithms to predict the possible interactions between *Ae. aegypti* cellular miRNAs and three arboviruses. We further sorted the results according to the sequence conservation among genotypes or the miRNA-vRNA interactions with low MFE and provided several candidates for further investigation. Even though we could merely provide a list of candidates, it remains necessary to evaluate the role of each potential miRNA on virus replication, and the subsequent reduction or facilitation of virus replication has to be demonstrated experimentally. Inhibitor studies should shed light on individual miRNA-vRNA interactions. However, miRNA-vRNA interactions can also be proven by techniques such as Argonaute-crosslinking immunoprecipitation (AGO-CLIP), and mutagenic analysis of virus genomes is possible for many of the arboviruses investigated here.

## Supplementary Materials

The following are available online at https://www.mdpi.com/1999-4915/11/6/540/s1, Figure S1: Venn-diagram presentation of common prediction binding sites on CHIKV negative sense RNA genome of each genotype/lineage., Table S1: Most common miRNA sites targeting each genotype of CHIKV, DENV1-4, and ZIKV, Table S2: Most common miRNA sites targeting each genotype of negative-sense CHIKV RNA genome.
