# Supplementary material for: Assessing the Potential Interactions between Cellular miRNA and Arboviral Genomic RNA in the Yellow Fever Mosquito, Aedes aegypti
Source: Viruses. 2019 Jun 10;11(6):540. doi: 10.3390/v11060540 (PMC6631873; doi:10.3390/v11060540)
Supplement: Supplementary file 1 [file viruses-11-00540-s001.zip › SUPPL/MS Yen_miRNA Article_Supplementary Table 1.pdf]

**Assessing the potential interactions between cellular miRNA and arboviral genomic RNA in the Yellow Fever mosquito, *Aedes aegypti***

**Pei-Shi YEN[a], Chun-Hong CHEN[b], Vattipally SREENU[c], Alain KOHL[c] and Anna-Bella FAILLOUX[a]**

**Supplementary Table 1** Most common miRNA sites targeting each genotype of CHIKV, DENV1-4, and ZIKV.

| miRNAs | viruses | virus genotypes | binding regions | sequences | MFE (kcal/mol, miRanda) | Regulation/Description |
|---|---|---|---|---|---|---|
| aae-miR-1-3p | DENV-1 | I | Capsid | UUUCAUAGCAUUCCUAACAUUUC | -13.06 | • Upregulated in female fat body at 24 post-blood meal[89]<br>• Significantly enriched at 14 days post-infection in ZIKV-infected mosquitoes[29] |
| | | | NS3 | AGACGAGGAAAGAGACAUUCC | -9.86 | |
| | | II | NS3 | AGAUGAGGAAAGAGACAUUCC | -8.99 | |
| | | III | Capsid | GGUGAUGGCUUUUAUAGCAUUCC | -12.26 | |
| | | | NS3 | AGAUGAGGAAAGAGACAUUCC | -8.99 | |
| | | IV | Capsid | GGUGAUGGCUUUUAUAGCAUUCC | -12.26 | |
| | | | NS3 | AGAUGAGGAAAGAGACAUUCC | -8.99 | |
| | | V | NS3 | AGAUGAGGAAAGAGACAUUCC | -8.99 | |
| | DENV-2 | Asian-II | NS2B | CAGUUCACUUCUAAAGAAUGACAUUCC | -13.73 | |
| | | American | E | AGGUUUGUCUGCAAACAUUCC | -12.96 | |
| | DENV-4 | I | NS5 | AAAGGUGAGGAAAGACAUUCC | -11.89 | |
| | | II | NS5 | AAAGGUGAGGAAAGACAUUCC | -11.89 | |
| | | III | NS5 | AAAAGUAAGGAAAGACAUUCC | -11.6 | |
| aae-miR-11-5p | CHIKV | IOL | 26S RNA UTR | AUCAGCUACAAUGGAGUUC | -18.84 | • Detectable during all developmental stages[90]<br>• Upregulated in the midgut after blood meal[91]<br>• *Wolbachia*-suppressed miRNA[92] |
| | | ECSA | 26S RNA UTR | AUCAGCUACAAUGGAGUUC | -18.84 | |
| | DENV-1 | I | NS1 | CACUCUAUGGAGCAAUGGAGUUC | -19.64 | |
| | | | NS4B | AGAUGGAUAUAGGAGUUC | -13.28 | |
| | | II | NS1 | CACUCUAUGGAGCAAUGGAGUUC | -19.64 | |
| | | | NS4A | CCUCCAUCAUACUGGAGUUC | -22.08 | |
| | | | NS4B | AGAUGGACAUAGGAGUUC | -14.12 | |
| | | IV | NS4B | AGAUGGACAUAGGAGUUC | -14.12 | |
| | | V | NS4B | AGAUGGACAUAGGAGUUC | -14.12 | |

| | | | | | | |
|---|---|---|---|---|---|---|
| | DENV-2 | Asian-II | NS5 | GAGAAGAAGCUAGGGGAGUUC | -13.41 | |
| | | American | NS4A | CUUCAAUAAUAUUGGAGUUC | -15.89 | |
| | DENV-3 | II | NS2A | AACACAUGAUUGCAGGGGUUC | -18.19 | |
| | | V | NS2A | AACACAUGAUUGCAGGGGUUC | -18.19 | |
| | DENV-4 | I | NS5 | CUCAUUUCGAGAGUGAAGGAGUUC | -18.12 | |
| | | II | E | UUCAGGAAAGGGAGUUC | -17.03 | |
| | | | NS5 | CUUCAGAGAGUGAAGGAGUUC | -18.25 | |
| | ZIKV | EA | NS1 | UCCUAGAGGAGAAUGGAGUUC | -16.41 | |
| aae-miR-124-3p | DENV-1 | II | NS5 | UGAUUGGCAACAAGUGCCUU | -14.07 | • Detectable during the larval stage[90] |
| | | IV | NS5 | CGAUUGGCAACAAGUGCCUU | -14.07 | |
| | DENV-2 | Asian American | NS5 | CGAUUGGACACAAGUGCCUU | -16.29 | |
| | DENV-3 | I | NS4A | AAAUAGGAAGAGUGCCUU | -10.41 | |
| | | II | NS4A | AAAUAGGAAGAGUGCCUU | -10.41 | |
| | | III | NS4A | AAAUAGGAAGAGUGCCUU | -10.41 | |
| | | V | NS4A | AAAUAGGAAGAGUGCCUU | -10.41 | |
| aae-miR-219-5p | DENV-1 | IV | E | AUGAGAUGGUGUUGUUGACAAUG | -15.76 | • *Wolbachia* induced miRNA in *Ae. aegypti* Aag2 cells[92] • Detectable during all developmental stages[90] |
| | DENV-2 | Asian-I | NS2A | AUUGGAUACCAUUAGCACUGACAAUC | -10.77 | |
| | | Asian-II | NS3 | GGAAAAAAGCAUUGAAGACAAUC | -11.9 | |
| | | American | NS3 | UGAAAAAAGCAUUGAAGACAAUC | -11.9 | |
| | | Cosmopolitan | NS3 | CUGAAAAAAACAUUGAAGACAAUC | -8.93 | |
| | DENV-3 | I | 2K | AGAGAACUCCCCAAGACAAUC | -12.56 | |
| | DENV-4 | I | 2K | CAAAGGACCCCACAAGACAAUC | -12.61 | |
| | | | NS5 | AGAAAAACCAGACAUGACAAUC | -10.25 | |
| | | II | 2K | CAAAGGACCCCACAAGACAAUC | -12.61 | |
| | | | NS5 | AGAAAAACCAGACAUGACAAUC | -10.25 | |
| | | III | NS5 | AGAGAAACCAGACAUGACAAUC | -12.86 | |
| | ZIKV | WA | NS4A | UCAGGAAGCCAUUGACAAUC | -14.09 | |
| aae-miR-263a-3p | DENV-1 | I | 3'UTR | AUCAUUCCAGGCACAGAACGC | -18.04 | • Detectable in mosquito cells [92] |
| | | II | NS2A | AAAAUGCUGAUGGUUGGAACAC | -15.5 | |

| | | | | | | |
|---|---|---|---|---|---|---|
| | | III | 3'UTR | AUCAUUCCAGGCACAGAACGC | -18.04 | |
| | | IV | 3'UTR | AUCAUUCCAGGCACAGAACGC | -18.04 | |
| | | V | 3'UTR | AUCAUUCCAGGCACAGAACGC | -18.04 | |
| | DENV-2 | Asian-I | 3'UTR | AUCAUUCCAGGCACAGAACGC | -18.04 | |
| | | Asian American | 3'UTR | AUCAUUCCAGGCACAGAACGC | -18.04 | |
| | | American | 3'UTR | AUCAUUCCAGGCACAGAACGC | -18.04 | |
| | | Cosmopolitan | 3'UTR | AUCAUUCCAGGCACAGAACGC | -18.04 | |
| | DENV-3 | II | NS2B | AUGAUGAGACUGAGAACAU | -17.25 | |
| | | | 3'UTR | AUCAUUCCAGGCACAGAACGC | -18.04 | |
| | | III | NS2B | AUGAUGAGACUGAGAACAU | -17.25 | |
| | | | 3'UTR | AUCAUUCCAGGCACAGAACGC | -18.04 | |
| | | V | 3'UTR | AUCAUUCCAGGCACAGAACGC | -18.04 | |
| | DENV-4 | III | NS2B | UAUAUGUGGCAAGUGAGAACAC | -15.62 | |
| | ZIKV | WA | NS5 | GUGGACAAGGAAAGAGAACAC | -12.13 | |
| aae-miR-263a-5p | CHIKV | IOL | nsP3 | AAUGCUAUUUGACCACAACGUGCCAU | -15.1 | • Increased in the saliva of CHIKV-infected mosquitoes at 10 days post-infection[47] |
| | | WA | E2 | UGCAAAAUUGAUCAGUGCCAU | -16.75 | |
| | | Asian | nsP3 | AUGUUAUUUGAUCACAAUGUGCCAU | -14.78 | |
| | | ECSA | nsP3 | AAUGCUAUUUGACCACAACGUGCCAU | -15.1 | |
| | DENV-1 | II | NS3 | GAGAUAGUUGACCUUAUGUGCCAU | -13.31 | |
| | DENV-2 | Cosmopolitan | NS3 | GAGUGGAACAUAUGUGAGUGCCAU | -17.18 | |
| | ZIKV | EA | NS3 | UCGUUGAUUUGAUGUGCCAU | -16.78 | |
| | | WA | NS3 | UCGUCGAUUUGAUGUGCCAU | -16.78 | |
| aae-miR-279-3p | CHIKV | IOL | E1 | AGUACCGUAUAAGACUCUAGUC | -12.34 | • Present in embryos, and could be induced in the midgut after blood meal[91]<br>• In Drosophila, miR-279-3p is involved in the formation of the $CO_2$ sensory neuron in maxillary palps[93] |
| | | Asian | E1 | AGUACCGUAUAAGACUCUAGUC | -12.34 | |
| | | ECSA | E1 | AGUACCGUAUAAGACUCUAGUC | -12.34 | |
| | DENV-1 | III | NS1 | AGAAGGAAGAGAAUCUAGUC | -14.82 | |
| | | V | NS1 | AGAAGGAGGAGAAUCUAGUC | -17.52 | |
| | ZIKV | Asian | NS5 | UAGGUGAGUCAUCAUCUAGUC | -18.35 | |
| | | EA | NS5 | UAGGUGAGUCAUCAUCUAGUC | -18.35 | |

| | | | | | | |
|---|---|---|---|---|---|---|
| aae-miR-281-3p | CHIKV | IOL | nsP1 | GUUCAUGUACAAUGCCAUGGC | -16.96 | • Detectable in Aag2 cells[92]<br>• Upregulated in the midgut after blood meal[91] |
| | | Asian | nsP3 | CCUUUGCCGUUAUGCCAUGAC | -11.74 | |
| | DENV-2 | Asian-I | NS2B | AAAAAAUGAUAUCCCCAUGAC | -13.59 | |
| | | | NS5 | UGUUCACACCAUUUCCAUGAG | -12.5 | |
| | | Asian-II | NS2B | UAAAGAAUGACAUUCCCAUGAC | -18.78 | |
| | | Asian American | NS2B | UAAAGAAUGACAUUCCUAUGAC | -18.78 | |
| | | Cosmopolitan | NS2B | UAAAGAAUGAUAUUCCCAUGAC | -18.7 | |
| | DENV-3 | I | prM | GCUGGUCACCCCAUCCAUGAC | -14.86 | |
| | | II | prM | GCUGGUCACUCCAUCCAUGAC | -16.07 | |
| | | III | prM | GCUGGUCACCCCAUCCAUGAC | -14.86 | |
| | | V | prM | GCUGGUUACCCCAUCCAUGAC | -14.86 | |
| | DENV-4 | I | NS2A | UGGUAAUAGGAAUGGCCAUGAC | -17.21 | |
| | | II | E | GGAACACUUCAAUGGCCAUGAC | -17.44 | |
| | | II | NS2A | UGGUAAUAGGAAUGGCCAUGAC | -17.21 | |
| | | III | NS2A | UGGUGAUAGGAAUGGCCAUGAC | -18.91 | |
| aae-miR-282-5p | DENV-1 | I | Capsid | CCCCCAACAGCAGGAAUUUUGGCUAGAU | -19.16 | • The expression profile is not clear |
| | | | NS3 | AAGAAAGAAACUACGACCCCGCUGGCUAGAU | -16.91 | |
| | | II | Capsid | CCCCCAACAGCAGGAAUUUUGGCUAGAU | -19.16 | |
| | | | E | GAUUGUUCACCUAGAACAGGGCUAGAU | -23.42 | |
| | | III | Capsid | CCCCCAACAGCAGGAAUUUUGGCUAGAU | -19.16 | |
| | | IV | Capsid | CCUCCAACAGCAGGAAUUUUGGCUAGAU | -19.16 | |
| | | V | Capsid | CCCCCAACAGCAGGAAUUUUGGCUAGAU | -19.16 | |
| | DENV-3 | II | Capsid | CCACCAACAGCAGGAGUUUUGGCUAGAU | -23.63 | |
| | | III | Capsid | CCACCAACAGCAGGAGUCUUGGCUAGAU | -23.63 | |
| | ZIKV | Asian | NS3 | AGAGUAAUGACUCGUAGACUGCUAGGU | -18.21 | |
| aae-miR-285-5p | CHIKV | IOL | E1 | GCACUGAUUCUAAUCGUGGUGCU | -22.74 | • Detectable in both male and female adults, but rarely observed in the larval stage[90] |
| | | WA | E1 | GCCUUAAUUUUAAUUGUGGUGCU | -14.41 | |
| | | ECSA | E1 | GCACUGAUUCUAAUCGUGGUGCU | -22.74 | |
| | DENV-2 | Cosmopolitan | NS3 | ACGCAGACAGAAGAUGGUGCU | -13.33 | |

| | | | | | | |
|---|---|---|---|---|---|---|
| | DENV-3 | II | NS3 | ACACAGAUAGAAAAUGGUGCU | -15.32 | |
| | ZIKV | WA | NS1 | GGGUCAUAGAGGAAUGGUGCU | -18.44 | |
| | | | NS3 | ACACAGAUAGAAGAUGGUGCU | -14.94 | |
| | | Asian | NS3 | ACACAGAUAGAAGAUGGUGCU | -14.94 | |
| aae-miR-286a-3p | CHIKV | ECSA | E1 | ACGGUGGGAGUACCGUAUAAGACUCUAGUC | -22.09 | • Significantly reduced at 2 days after ZIKV infection[29]<br>• Detectable during embryo stage, and less abundant in adults[90,91]<br>• Ovary specific expressed miRNA [94] |
| | ZIKV | WA | 5'UTR | UGGAAACGAGAGUUUCUGGUC | -23.15 | |
| | | | NS5 | AUGAUCCAAGAUUUUGGGCUCUAGUG | -13.39 | |
| | | Asian | 5'UTR | UGGAAACGAGAGUUUCUGGUC | -23.15 | |
| | | | NS5 | CAUAGGUGAGUCAUCAUCUAGUC | -18.44 | |
| | | EA | 5'UTR | UGGAAACGAGAGUUUCUGGUC | -23.15 | |
| | | | NS5 | CAUAGGUGAGUCAUCAUCUAGUC | -18.44 | |
| aae-miR-286b-3p | CHIKV | ECSA | E1 | ACGGUGGGAGUACCGUAUAAGACUCUAGUC | -20.23 | • Significantly reduced at 7 and 14 days after ZIKV infection[29]<br>• Ovary specific expressed miRNA [94] |
| | DENV-1 | I | NS2A | UUCUUACAAUUGGAUUGAGUCUAGUG | -14.43 | |
| | ZIKV | WA | 5'UTR | UGGAAACGAGAGUUUCUGGUC | -24.83 | |
| | | Asian | 5'UTR | UGGAAACGAGAGUUUCUGGUC | -24.83 | |
| | | EA | 5'UTR | UGGAAACGAGAGUUUCUGGUC | -24.83 | |
| | | | NS5 | CAUAGGUGAGUCAUCAUCUAGUC | -16.72 | |
| aae-miR-305-5p | CHIKV | IOL | nsP2 | AAUGCGCACUACGAAUGAGUACAA | -14.17 | • Abundant in Aag2 cells and mosquitoes during the pupal stage[29,90,92]<br>• Upregulated in salivary glands after CHIKV infection[47]<br>• The expression switched to aae-miR-305-3p in response to DENV-2 infection[25]<br>• Upregulated in the fat body of *Ae. aegypti* after blood meal[95]<br>• Down regulated in response to DENV-2 infection[31] |
| | | | E1 | UGAUUACAUCACGUGCGAGUACAA | -14.81 | |
| | | Asian | nsP2 | AAUGCGCACUACGAAUGAGUACAA | -14.17 | |
| | | ECSA | nsP2 | AAUGCGCACUACGAAUGAGUACAA | -14.17 | |
| | DENV-3 | III | E | AUACUCAUUAAGGUUGAGUACAA | -11.67 | |
| | DENV-4 | I | NS1 | CGUGCACACUUGGACAGAACAGUACAA | -16.46 | |
| | | | NS5 | GGAAGGAGUUUGAAGAGUACAA | -14.83 | |
| | | | NS5 | CACAUUCACCAACAUGGAAGUACAA | -17.17 | |
| | | II | NS1 | CGUGCACACUUGGACAGAACAGUACAA | -16.46 | |
| | | III | NS5 | CACAUUCACCAACAUGGAAGUACAA | -17.17 | |
| aae-miR-315-5p | CHIKV | IOL | nsP1 | UCCCUUUGAGGACUAGAAUCAAA | -11.73 | • Induced by Palm Creek virus (PCV) infection at 6 days post-infection[30]<br>• Potentially targets the 3'UTR of the juvenile hormone-regulated |
| | | WA | nsP4 | AAAAUUUGCGUGCAAUCAAG | -12.55 | |

| | | | | | | |
|---|---|---|---|---|---|---|
| | | ECSA | nsP1 | UCCCGUUGAGGACUAGAAUCAAA | -11.13 | serine protease (JHA15) and AaArgM3[96,97] |
| | DENV-1 | IV | NS5 | ACCAAUUGAGCAAAUCAGA | -16.2 | |
| | DENV-2 | Asian-I | Capsid | GGGAACAAUUAAAAAAUCAAA | -11.23 | |
| | | Asian-II | NS5 | AUCCAAACAGCAAUAAAUCAAG | -11.95 | |
| | | American | NS5 | UAGUUCCAUGCAGAAAUCAAG | -13.29 | |
| | | Cosmopolitan | NS5 | AUCCAAACAGCAAUAAAUCAAG | -11.95 | |
| | ZIKV | EA | Capsid | CCAUGUUGAGAAUAAUCAAU | -14.27 | |
| | | WA | 2K | AUCUCCCCAGGAUAAUCAAA | -9.35 | |
| aae-miR-316-5p | CHIKV | IOL | nsP1 | GCCCGGGACGCAGAAAAAGAA | -13.83 | • Detectable in the fat body[95]<br>• Upregulated in midgut, 24 hrs after blood meal[91] |
| | DENV-1 | I | Capsid | GAACAACCAACGGAAAAAGAC | -20.34 | |
| | | | NS5 | AUGGGAAAAGUGAGGAAAGAC | -19.3 | |
| | | II | NS3 | GGCCAGUGUCAAAAAAGAC | -14.4 | |
| | | | NS3 | AUCCAAGAUGAGGAAAGAGAC | -18 | |
| | | | NS5 | AUGGGAAAAGUAAGAAAAGAC | -17.89 | |
| | | III | Capsid | GAACAACCAACGGAAGAAGAC | -20.48 | |
| | | | NS5 | UGGGAGAAAAAUGGAAAAGAC | -19.12 | |
| | | | NS5 | AUGGGAAAAGUGAGAAAAGAC | -19.68 | |
| | | IV | Capsid | GAACAACCAACGGAAAAAGAC | -20.34 | |
| | | V | Capsid | GAACAACCAACGGAAAAAGAC | -20.34 | |
| | | | NS5 | AUGGGAAAAGUAAGAAAAGAC | -17.89 | |
| | DENV-2 | Asian-I | Capsid | GAAUAACCAACGGAAAAAGGC | -19.59 | |
| | | | NS5 | AAGAAUUAGGGAAGAAAAAGAC | -13.98 | |
| | | Asian-II | Capsid | GAAUAACCAACGGAAAAAGGC | -19.59 | |
| | | Asian American | Capsid | GAAUAACCAACGGAAAAAGGC | -19.59 | |
| | | | NS1 | UGUCAGCAGCCAUAAAAGAC | -15.36 | |
| | | American | Capsid | GAAUAACCAACGGAAAAAGGC | -19.59 | |
| | | | NS5 | AAGAACUAGGAAAGAAAAAGAC | -15.04 | |
| | | | NS5 | CAACAUGAUGGGAAAAAGAG | -13.52 | |
| | | Cosmopolitan | Capsid | GAAUAACCAACGGAAAAAGGC | -19.59 | |

| | | | | | | |
|---|---|---|---|---|---|---|
| | | | NS1 | UGUCAGCAGCCAUAAAAGAC | -15.36 | |
| | | | NS4A | UCUUGAUGAGCGGAAAAGGC | -20.74 | |
| | DENV-3 | I | Capsid | GAACAACCAACGGAAAAAGAC | -20.34 | |
| | | | NS3 | UGACACAGAAUAUCAAAAGAC | -8.61 | |
| | | II | Capsid | GAACAACCAACGAAAAAAGAC | -13.47 | |
| | | | Capsid | UAUCAACAAAAGGAAAAAGAC | -17.7 | |
| | | III | Capsid | GAACAACCAACGGAAGAAGAC | -20.48 | |
| | | | Capsid | AAUCAACCAACGGAAAAAGAC | -20.4 | |
| | | V | Capsid | GAACAACCAACGGAAAAAGAC | -20.34 | |
| | | | Capsid | UAUCAACAAACGGAAAAAGAC | -20.4 | |
| | DENV-4 | I | NS5 | GAACAGAGUGUGGAUAGAAGAC | -21.5 | |
| | | II | NS5 | GAACAGAGUGUGGAUAGAAGAC | -21.5 | |
| | ZIKV | EA | NS5 | AGUCCUGAAGUGGAAGAGAC | -18.52 | |
| aae-miR-34-3p | CHIKV | IOL | nsP1 | CAUGCCGCUGUGAUACAGUGGUU | -14.18 | • Upregulated in response to CHIKV infection[53]<br>• Detectable during all the developmental stages, but the expression level was relatively low during the pupal stage[90]<br>• The expression of this miRNA was correlated with the nutritional status of adults and can be suppressed in the midgut when adults are starved[98]<br>• Increased in the abundance of 5′ Trim isomiRs in response to DENV-2 infection[99] |
| | | | nsP1 | GCUGAACCAGAGAAUAGUGGUU | -14.74 | |
| | | WA | nsP4 | GUCCAAAGGCUGAAGGGUGGUU | -19.31 | |
| | | Asian | nsP1 | CAUGCCGCUGUGACACAGUGGUU | -14.18 | |
| | | | nsP1 | GCUGAACCAGAGAAUAGUGGUU | -14.74 | |
| | | ECSA | nsP1 | CAUGCCGCUGUGAUACAGUGGUU | -14.18 | |
| | | | nsP1 | GCUGAACCAGAGAAUAGUGGUU | -14.74 | |
| | DENV-1 | I | 3'UTR | CAUGGGGUAGCAGACUAGUGGUU | -20.16 | |
| | | III | 3'UTR | CAUGGGGUAGCAGACUAGUGGUU | -20.16 | |
| | | IV | 3'UTR | CAUGGGGUAGCAGACUAGUGGUU | -20.16 | |
| | DENV-2 | Asian-I | NS2A | AGACCUAGGAAGAGUGGUGGUU | -16.15 | |
| | | Cosmopolitan | NS5 | UCAUCCAUGGUGAACGGAGUGGUU | -18.17 | |
| | DENV-3 | I | prM | UUCCUUGACCCAGAAAGUGGUU | -10.1 | |
| | | | 3'UTR | CACGGUGUAGCAGACUAGUGGUU | -20.21 | |
| | | II | prM | UUCCCUGACCCAGAAAGUGGUU | -10.1 | |
| | | | NS3 | GACUGUAUGGCAAUGGAGUGGUU | -19.15 | |

| | | | | | | |
|---|---|---|---|---|---|---|
| | | III | NS3 | GACUGUAUGGCAAUGGAGUGGUU | -19.15 | |
| | | V | prM | UUCCUUGACCCAGAAAGUGGUU | -10.1 | |
| | | | NS3 | GACUGUAUGGCAAUGGAGUGGUU | -19.15 | |
| | | | 3'UTR | CACGGUGUAGCAGACUAGUGGUU | -20.21 | |
| | DENV-4 | I | E | GUGAACAAGGAAAAAGUGGUU | -17.78 | |
| | | II | E | GAACCUUGAAUACACAGUGGUU | -13.34 | |
| | | III | E | UGUGAACAAAGAAAAAGUGGUU | -14.92 | |
| | | | NS5 | CAAAACUAGGCACACGAGUGGUU | -15.39 | |
| | ZIKV | WA | 3'UTR | UCCAGCAGAAGGACUAGUGGUU | -20.97 | |
| | | Asian | 3'UTR | CCCAGAAGAGGGACUAGUGGUU | -17.3 | |
| | | EA | E | GUUGGUGCACAAAGAGUGGUU | -13.53 | |
| | | | 3'UTR | UCCAGCAGAGGGACUAGUGGUU | -21.41 | |
| aae-miR-79-3p | DENV-1 | I | NS4A | AAACGUUAAUGCUCCUAGCUUU | -13.74 | • Relatively low expression level[91] |
| | | III | NS4A | AAACAUUGAUGCUCCUAGCUUU | -13.75 | |
| | | IV | 2K | CAUAUGUGGUGAUAGCUUU | -15.19 | |
| | | V | NS4A | AAACAUUGAUGCUUCUAGCUUU | -16.32 | |
| | DENV-3 | I | Capsid | UGGUCAUGGCGUUCAUAGCUUU | -11.8 | |
| | | II | Capsid | UGGUUAUGGCGUUCAUAGCUUU | -14 | |
| | | III | Capsid | UGGUUAUGGCGUUCAUAGCUUU | -14 | |
| | | V | Capsid | UGGUUAUGGCGUUUAUAGCUUU | -12.3 | |
| | DENV-4 | II | prM | AGAGAAGCGCUCAGUAGCUUU | -11.81 | |
| aae-miR-92a-3p | DENV-1 | I | NS5 | GCAAAAGGAAGUCGUGCAAU | -13.7 | • The expression profile is not clear |
| | | II | NS5 | GCAAAAGGAAGUCGUGCAAU | -13.7 | |
| | | III | NS5 | GCAAAAGGAAGUCGUGCAAU | -13.7 | |
| | | V | NS5 | GCAAAAGGAAGUCGUGCAAU | -13.7 | |
| | DENV-3 | I | prM | ACCUACGGAACGUGCAAU | -13.85 | |
| | | II | prM | GACUUAUGGAACGUGCAAU | -11.68 | |
| | | III | prM | GACUUAUGGAACGUGCAAU | -11.68 | |
| | | | E | AAUAGAGGGAAAAGUGGUGCAAU | -19.35 | |

| | | | | | | |
|---|---|---|---|---|---|---|
| aae-miR-92b-3p | DENV-1 | I | NS5 | GGCAAAAGGAAGUCGUGCAAU | -14.72 | • The expression profile is not clear |
| | | II | NS5 | GGCAAAAGGAAGUCGUGCAAU | -14.72 | |
| | | III | NS5 | GGCAAAAGGAAGUCGUGCAAU | -14.72 | |
| | | V | NS5 | GGCAAAAGGAAGUCGUGCAAU | -14.72 | |
| | DENV-2 | Asian-I | NS3 | UGGAGCAUAUGUGAGUGCAAU | -12.57 | |
| | DENV-3 | I | prM | GACCUACGGAACGUGCAAU | -14.11 | |
| | | II | prM | UGACUUAUGGAACGUGCAAU | -12.13 | |
| | | III | prM | UGACUUAUGGAACGUGCAAU | -12.13 | |
| | | | E | CAAUAGAGGGAAAAGUGGUGCAAU | -17.65 | |
| | DENV-4 | II | NS2B | GAAGGCCGCUAGUGUGCAAU | -20.74 | |
| | ZIKV | WA | NS5 | AAAGCAAAAGGCAGCCGUGCAAU | -14.29 | |
| aae-miR-989-3p | CHIKV | IOL | nsP4 | CAACACAUUGUUAAACAUCAC | -15.2 | • Down regulated in response to CHIKV infection in *Ae. aegypti* Aag2 cells[53]<br>• Up regulated in response to ZIKV infection at 2 and 14 days post-infection[29]<br>• Down regulated in *Wolbachia*-infected mosquitoes[100]<br>• The most abundant miRNA in the ovaries[94] |
| | | WA | nsP4 | AAUACACUGCUAAACAUCAC | -18.88 | |
| | | | E1 | CUGUCACUUGACUACAUCAC | -20.31 | |
| | | ECSA | nsP4 | CAACACACUGCUAAAUAUCAC | -17.71 | |
| | DENV-1 | II | NS5 | UCUAGAUUACAUGACAUCAA | -11.96 | |
| | | III | NS5 | UUUAGACUACAUGACAUCAA | -14.18 | |
| | | | NS4B | CUGAUACUUUGUACAUCAC | -18.69 | |
| | | V | NS4B | CUGAUACUUUGUACAUCAC | -18.69 | |
| | ZIKV | Asian | NS2B | GAAAGAGCAGGUGACAUCAC | -15 | |
| | | EA | NS2B | GAAAGAGCAGGUGACAUCAC | -15 | |
| aae-miR-996-3p | CHIKV | IOL | E1 | GAGUACCGUAUAAGACUCUAGUC | -15.27 | • The most abundant miRNA in embryos[94] |
| | | Asian | E1 | GAGUACCGUAUAAGACUCUAGUC | -15.27 | |
| | | ECSA | E1 | GAGUACCGUAUAAGACUCUAGUC | -15.27 | |
| | DENV-1 | III | NS1 | GAGAAGGAAGAGAAUCUAGUC | -16.47 | |
| | ZIKV | Asian | NS5 | GGUGAGUCAUCAUCUAGUC | -18.33 | |
| | | EA | NS5 | GGUGAGUCAUCAUCUAGUC | -18.33 | |
| aae-miR-998-3p | CHIKV | WA | E1 | CCUUAAUUUUAAUUGUGGUGCU | -16.67 | • Detectable during all the developmental stages[91] |
| | DENV-1 | I | 3'UTR | AGAAAAUGGAAUGGUGCU | -15.16 | |

| | | | | | | |
|---|---|---|---|---|---|---|
| | | III | 3'UTR | AGAAAAUGGAAUGGUGCU | -15.16 | |
| | | IV | 3'UTR | AGAAAAUGGAAUGGUGCU | -15.16 | |
| | | V | E | CUUUAAUGAGAUGGUGCU | -16.04 | |
| | | | 3'UTR | AGAAAAUGGAAUGGUGCU | -15.16 | |
| | DENV-2 | Asian-I | 3'UTR | AGAAAAUGGAAUGGUGCU | -15.16 | |
| | | Asian-II | 3'UTR | AGAAAAUGGAAUGGUGCU | -15.16 | |
| | | Asian American | 3'UTR | AGAAAAUGGAAUGGUGCU | -15.16 | |
| | | Cosmopolitan | 3'UTR | AGAAAAUGGAAUGGUGCU | -15.16 | |
| | DENV-3 | I | 3'UTR | AGAAAAUGGAAUGGUGCU | -15.16 | |
| | | II | NS3 | CAGAUAGAAAAUGGUGCU | -13.96 | |
| | | | 3'UTR | AGAAAAUGGAAUGGUGCU | -15.16 | |
| | | III | NS3 | CAGAUAGAAAAUGGUGCU | -13.96 | |
| | | | 3'UTR | AGAAAAUGGAAUGGUGCU | -15.16 | |
| | | V | NS3 | CAGAUAGAAAAUGGUGCU | -13.96 | |
| | | | 3'UTR | AGAAAAUGGAAUGGUGCU | -15.16 | |
| aae-miR-9c-5p | DENV-2 | Asian-I | NS5 | AUCAUAUCGAGAAGAGACCAAAG | -16.48 | • Down regulated in DENV-2 infected mosquitoes at 9 days post-infection[31] • Down regulated in the fat body of mosquitoes at 24 hrs after blood meal[95] |
| | | Asian-II | NS5 | AUCAUAUCGAGAAGAGACCAAAG | -16.48 | |
| | | Asian American | NS5 | AUCAUAUCGAGAAGAGACCAAAG | -16.48 | |
| | | American | NS5 | CAUAUCGAGAAAAGACCAAAG | -13.98 | |
| | | Cosmopolitan | NS5 | AUUAUAUCGAGAAGAGACCAAAG | -18.63 | |
| | DENV-4 | I | 3'UTR | UCACCAACAAAAACACCAAAG | -11.81 | |
| | | II | 3'UTR | CCAACAACAAACACCAAAG | -11.47 | |
| | | III | Capsid | GGGACAGUUGAAGAAAACCAAGG | -19.29 | |
| | | | NS5 | AUCAUAUCCAGGAAAGACCAAAG | -14.95 | |
| | ZIKV | Asian | NS5 | AUUAUUUCGAGACAAGACCAAAG | -14.59 | |
| aae-miR-iab-4-5p | CHIKV | IOL | nsP1 | CCAUCAUGGAUCCUGUGUACG | -16.87 | • Arm switched from 5’ to 3’ in response to DENV-2 infection[25] |
| | | | 3'UTR | GAAAAAUACAUAAAUAGGUAUACG | -16.31 | |
| | | WA | 3'UTR | AAAUCUCAUAAACAGGUAUACG | -12.6 | |
| | | ECSA | nsP1 | CCAUCAUGGAUCCUGUGUACG | -16.87 | |

| | | | | | | |
|---|---|---|---|---|---|---|
| | | | 3'UTR | GAAAAAUACAUAAAUAGGUAUACG | -16.31 | |
| | DENV-2 | Asian-II | prM | UGAAGAUACAAUCACGUAUAAG | -15.14 | |
| aae-miR-new8 | CHIKV | IOL | nsP1 | GGCAGGUCACACCGAAUGACCA | -10.63 | • Detectable in the very early stages of embryo development and decreases during embryogenesis[90] |
| | | WA | nsP1 | GGCAGGUCACACCGAAUGACCA | -10.63 | |
| | DENV-1 | IV | NS1 | UUUACUCAAGUGUGUGACCA | -16.85 | |
| | | | NS2A | UGCAUGGAAGACAAUGGCCA | -13.16 | |
| | DENV-2 | Asian-I | NS2A | CCAAGGAAUUGAUGAUGACCA | -10.42 | |
| | | | NS3 | CCAAAAAAUGAAAAUGACCA | -15.73 | |
| | | Asian-II | NS5 | ACAUCAUGGCACUAUGACCA | -11.03 | |
| | | Asian American | NS3 | CCAAAAAAUGAAAAUGACCA | -15.73 | |
| | | | NS5 | ACAUCAUGGCACUAUGACCA | -11.03 | |
| | | Cosmopolitan | NS3 | CCAAAAAAUGAAAAUGACCA | -15.73 | |
| | | | NS5 | ACAUCAUGGCACUAUGACCA | -11.03 | |
| | DENV-3 | I | NS3 | CCACAAAAAGAGAAUGACCA | -16.53 | |
| | | II | NS5 | UCACCAUCAGUGGAUGACCA | -11.19 | |
| | | III | NS3 | CCACAAAAAGAGAAUGACCA | -21.12 | |
| | DENV-4 | I | NS5 | UACACGAGUAGUCAUGACCA | -15.34 | |
| | | II | NS2A | CUAAUGGUAAUAGGAAUGGCCA | -12.48 | |
| | | | NS5 | CACACGAAUGGUUAUGACCA | -15.44 | |
| | | | NS5 | CAUCACCAAUGGAUGACCA | -10 | |
| | | III | NS5 | CACACGAGUGGUUAUGACCA | -15.44 | |
| | ZIKV | WA | NS2A | UUUGAAGAAGAGAAUGACCA | -16.46 | |
| | | Asian | NS2A | GCUGAAGAAGAGAAUGACCA | -16.46 | |
| | | | NS5 | GGAAAGGGAGAAUGGAUGACCA | -13.49 | |
| | | EA | E | GUGUUCUAAGAAGAUGACCG | -18.86 | |
| | | | NS2A | GUUGAAGAAGAGAAUGACCA | -16.46 | |
| | | | NS5 | GGAAAGGGAGAAUGGAUGACCA | -13.49 | |
