# Supplementary material for: Assessing the Potential Interactions between Cellular miRNA and Arboviral Genomic RNA in the Yellow Fever Mosquito, Aedes aegypti
Source: Viruses. 2019 Jun 10;11(6):540. doi: 10.3390/v11060540 (PMC6631873; doi:10.3390/v11060540)
Supplement: Supplementary file 1 [file viruses-11-00540-s001.zip › SUPPL/MS Yen_miRNA Article_Supplementary Table 2.pdf]

Supplementary Table 2 Most common miRNA sites targeting each genotype of negative-sense CHIKV RNA genome.

miRNAs	virus genotypes	binding regions (negative sense)	sequences	MFE (kcal/mol, miRanda)
aae-miR-263a-3p	IOL	NS1	UUUAUAGCUAGAUGCGAGAACGC	-18.42
	Asian		UUUAUGGCUAGAUGCGAGAACGC	-18.59
	ECSA		UUUAUAGCUAGAUGCGAGAACGC	-18.42
aae-miR-278-3p	IOL	E2	CUACUGUCAGUGAAUCCCACCG	-19.82
	Asian		UACCAUCAGUGAACCCCACCG	-18.11
	ECSA		CUACUGUCAGUGAAUCCCACCG	-19.82
aae-miR-282-5p	IOL	NS1	CUGCUCUAUUAGUUUUAUAGCUAGAU	-11.32
	Asian		CUGCUCUAUUAGUUUUAUGGCUAGAU	-11.32
	ECSA		CUGCUCUAUUAGUUUUAUAGCUAGAU	-11.32
	WA		CUGCUCUAUUAGUUUUAUAGCUAGAU	-11.32
aae-miR-308-3p	IOL	E2	UGGCCAUUGACUGUGAU	-9.75
	Asian		CUGACUAUUGACUGUGAU	-11.28
	ECSA		UGGCCGUUGACUGUGAU	-9.75
aae-miR-79-3p	IOL	E2	UAAGUAUGGUCUUGUGGCUUU	-14.57
	ECSA		UAAGUAUGGUCUUGUGGCUUU	-14.57
	WA		UAGAUAUGGUCUUGUGGCUUU	-14.57
aae-miR-996-5p	IOL	NS3	CCCACCUGCCCUGUCUAGUC	-13.85
	Asian		CCCACCUGCCCUGUCUAGUC	-13.85
	ECSA		CCCACCUGCCCUGUCUAGUC	-13.85
aae-miR-iab-4-3p	IOL	3'UTR	AAUGUGUCUCUUAGGGGACACGUAUACC	-20.15
	ECSA		AAUGUGUCUCUUAGGGGACACGUAUACC	-20.15
	WA		AAUGUGUCUCUUAGGGGACACGUAUACC	-20.15
aae-miR-new17[1]	IOL	NS2	UCGUAGACGUACUCGUGUUCUGU	-21.63
	Asian		UCGUAGACGUACUCGUGUUCUGU	-22.41
	ECSA		UCGUAGACGUACUCGUGUUCUGU	-21.63

1 Hu, W., Criscione, F., Liang, S. & Tu, Z. MicroRNAs of two medically important mosquito species: Aedes aegypti and Anopheles stephensi. *Insect Mol Biol* **24**, 240-252, doi:10.1111/imb.12152 (2015).
