# Supplementary material for: Assessing the Potential Interactions between Cellular miRNA and Arboviral Genomic RNA in the Yellow Fever Mosquito, Aedes aegypti
Source: Viruses. 2019 Jun 10;11(6):540. doi: 10.3390/v11060540 (PMC6631873; doi:10.3390/v11060540)
Supplement: Supplementary file 1 [file viruses-11-00540-s001.zip › SUPPL/Supplementary Figure.pdf]

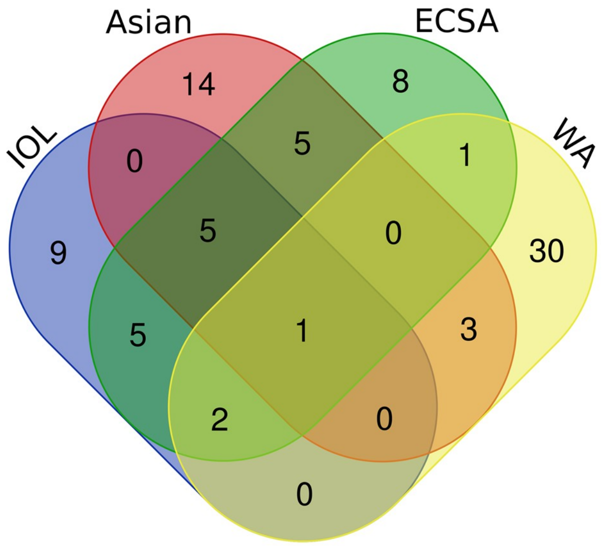

Supplementary Figure 1. Venn-diagram presentation of common prediction binding sites on CHIKV negative sense RNA genome of each genotype/lineage. miRNAs that were sorted by the conservation of target sites, might show a more general miRNA-vRNA interaction. IOL, Indian Ocean Lineage, AM258992; WA, West African, HM045816; Asian, EU703762; ECSA, East/Central/South African, HM045811.
